# Precision nutrition in gastric cancer: current advances and future directions

**DOI:** 10.3389/fnut.2026.1844696

**Published:** 2026-06-11

**Authors:** Zhen Wang, Lingbo Ma, Jie Mao

**Affiliations:** Lanzhou University Second Hospital, Lanzhou, China

**Keywords:** gastric cancer, gut microbiota, immunonutrition, precision nutrition, sarcopenia

## Abstract

Patients with gastric cancer frequently experience malnutrition, weight loss, and sarcopenia from diagnosis through treatment and follow-up. These conditions are not solely attributable to inadequate intake but are closely related to systemic inflammation, metabolic reprogramming, treatment-related toxicities, and altered digestion and absorption after gastrectomy. This review summarizes the theoretical basis, assessment approaches, stage-specific intervention strategies, and current evidence limitations of precision nutrition in gastric cancer. It focuses on nutritional risk screening, diagnosis of malnutrition based on the Global Leadership Initiative on Malnutrition (GLIM) criteria, computed tomography (CT)-based body composition analysis, energy and protein provision, support pathways including oral nutritional supplements, enteral nutrition, and parenteral nutrition, as well as emerging areas such as immunonutrition, microbiota-targeted interventions, AI-assisted body composition analysis, and multi-omics integration. Current evidence suggests that precision nutrition in gastric cancer should remain grounded in standardized nutritional assessment and guideline-recommended supportive strategies. Prospective, multicenter studies are needed to clarify the benefits and scope of nutritional interventions across different nutritional phenotypes.

## 1 Introduction

Gastric cancer remains a major contributor to the global cancer burden, with a particularly high incidence in East Asia (1, 2). In recent years, advances in minimally invasive surgery, perioperative treatment, immunotherapy, and targeted therapy have shifted gastric cancer care from surgery alone toward multidisciplinary management (1, 3). However, progress in treatment strategies has not eliminated nutrition-related problems. Malnutrition, weight loss, and skeletal muscle loss remain common throughout diagnosis, treatment, and follow-up, and are closely associated with reduced treatment tolerance, delayed postoperative recovery, impaired quality of life, and poor prognosis (4–6). On the one hand, patients may experience prolonged inadequate intake due to anorexia, early satiety, obstruction, nausea and vomiting, digestive and absorptive dysfunction, and treatment-related toxicities. On the other hand, tumor burden, systemic inflammation, and metabolic reprogramming may accelerate fat mobilization and skeletal muscle protein breakdown (4, 7, 8). Therefore, gastric cancer-related malnutrition and sarcopenia should not be understood simply as consequences of insufficient intake, but rather as complex metabolic phenotypes arising from tumor–host interactions (4, 7, 8). Based on this understanding, nutritional management should be incorporated early into the comprehensive treatment of gastric cancer, rather than being used only as a remedial measure after marked weight loss has occurred (4).

In clinical practice, adequate energy and protein provision remains the cornerstone of nutritional support for patients with gastric cancer. This approach is essential for correcting inadequate intake, maintaining metabolic requirements, and supporting anticancer treatment, but it cannot fully address the heterogeneity of nutrition-related problems in this population (4). Conventional indicators such as BMI, body weight change, and serum albumin may fail to capture occult muscle loss, sarcopenic obesity, or inflammation-driven malnutrition (4, 7, 9, 10). In addition, tumor location, pathological subtype, disease stage, treatment modality, and host inflammatory status may influence dietary intake, metabolic demand, muscle depletion, and response to nutritional intervention (1, 7, 9). Therefore, nutritional management in gastric cancer should move beyond empirical supplementation toward standardized assessment, risk stratification, and dynamic monitoring (4, 10).

In this review, precision nutrition does not refer to a direct translation of individual molecular markers, such as HER2, PD-L1, MSI/dMMR, EBV, or CLDN18.2, into specific nutritional prescriptions. Rather, it represents an individualized strategy based on standard nutritional support, integrating nutritional risk screening, diagnosis based on the Global Leadership Initiative on Malnutrition (GLIM) criteria, body composition assessment, inflammatory status, treatment phase, and potentially multi-omics data to guide risk stratification, intervention selection, and response monitoring (1, 3, 4, 10, 11). At present, precision nutrition in gastric cancer remains primarily phenotype-driven and risk-oriented, rather than a mature form of molecularly guided nutritional therapy (3, 4, 11).

Against this background, this review examines the theoretical basis, clinical pathways, and translational boundaries of precision nutrition in gastric cancer. It focuses on gastric cancer-related inflammation, metabolic reprogramming, and mechanisms of cancer-associated muscle wasting (7, 8), and further summarizes nutritional risk screening, GLIM-based diagnosis of malnutrition, body composition and functional assessment, energy and protein requirements, micronutrient management, and nutritional support pathways, including oral nutritional supplements (ONS), enteral nutrition (EN), and parenteral nutrition (PN) (3, 4, 10, 12). Across different clinical settings, including pretreatment, neoadjuvant therapy, the perioperative period, postoperative recovery, and advanced or metastatic disease, this review outlines the corresponding priorities for nutritional intervention (3, 4, 12). It also discusses emerging directions, such as immunonutrition, microbiota-targeted interventions, AI-assisted body composition analysis, and multi-omics integration, while emphasizing the need to distinguish evidence-supported clinical strategies from exploratory approaches that remain at the hypothesis-generating stage (1, 4, 13–16).

## 2 Biological basis of gastric cancer-related nutritional deterioration

### 2.1 Inadequate intake, digestive and absorptive dysfunction, and treatment-related injury

Nutritional deterioration in patients with gastric cancer is rarely attributable to a single cause (1, 3, 4). The tumor itself may restrict food intake through gastric luminal obstruction, pyloric stenosis, early satiety, pain, reflux, nausea and vomiting, and chronic blood loss. After gastrectomy and gastrointestinal reconstruction, reduced gastric capacity, altered gastric emptying, dumping syndrome, bile reflux, diarrhea, and impaired coordination of pancreaticobiliary secretion may further compromise dietary intake and nutrient absorption (1, 12, 17). Treatment-related injury is also clinically relevant. Neoadjuvant therapy, perioperative stress, postoperative infection, adjuvant therapy, and gastrointestinal toxicities associated with systemic treatment for advanced disease may aggravate anorexia, mucosal injury, diarrhea, and inadequate protein intake (3, 12, 17, 18). Together, these factors expose patients to repeated risks of weight loss, hypoalbuminemia, and skeletal muscle depletion throughout diagnosis, treatment, and follow-up (4, 7, 8). Therefore, gastric cancer-related malnutrition should not be viewed simply as a consequence of eating less, but rather as a dynamic pathological process involving tumor burden, inflammatory responses, treatment-related injury, and metabolic reprogramming (4, 7, 8, 19).

### 2.2 Systemic inflammation and enhanced catabolism

In patients with gastric cancer, systemic inflammation is an important driver of nutritional deterioration and muscle wasting (8, 9, 20, 21). *Helicobacter pylori* infection, chronic gastric mucosal inflammation, remodeling of the tumor microenvironment, surgical trauma, and infectious complications may all induce the release of pro-inflammatory cytokines, including interleukin-6 (IL-6), tumor necrosis factor-alpha (TNF-*α*), and interleukin-1 beta (IL-1β), and activate signaling pathways such as JAK/STAT3 and NF-κB (8, 14, 20–22). The IL-6/JAK/STAT3 axis can promote the acute-phase response and increase C-reactive protein levels, while also affecting hepatic gluconeogenesis, lipid metabolism, and skeletal muscle protein homeostasis. The TNF-*α*/NF-κB pathway may impair insulin signaling, promote lipolysis, disrupt appetite regulation, and upregulate genes involved in protein degradation (8, 20–22). Unlike simple starvation, inflammation-driven wasting is often characterized by the coexistence of insufficient intake and increased energy expenditure, and conventional energy and protein supplementation may not fully prevent continued skeletal muscle loss (4, 8, 20, 21). These findings suggest that nutritional management in gastric cancer should not focus solely on intake, but should also incorporate assessment of inflammatory burden and muscle metabolic status (4, 9).

### 2.3 Molecular mechanisms of cancer-associated muscle wasting

Skeletal muscle loss is one of the most clinically important phenotypes of nutritional deterioration in gastric cancer. It is driven by an imbalance between increased muscle protein degradation and impaired protein synthesis. Under the influence of pro-inflammatory cytokines, tumor-derived metabolic factors, and stress hormones, FOXO transcription factors are activated and subsequently upregulate the muscle-specific E3 ubiquitin ligases MuRF1 and Atrogin-1, thereby activating the ubiquitin–proteasome system and accelerating myofibrillar protein degradation. NF-κB signaling may also enhance the transcription of genes involved in protein degradation and promote muscle atrophy. At the same time, inhibition of the PI3K/Akt/mTOR pathway reduces the efficiency of muscle protein synthesis and weakens the anabolic response of skeletal muscle to amino acids and nutritional intervention (8, 20, 21, 23). Therefore, gastric cancer-related sarcopenia is not simply a consequence of insufficient protein intake, but rather reflects the combined effects of inflammation, endocrine stress, inadequate nutritional substrates, and dysregulated intracellular signaling in muscle cells (7, 8, 20, 21, 23). If nutritional status is assessed only by body weight, BMI, or serum albumin, low muscle mass, myosteatosis, and impaired muscle function may be easily overlooked (5, 7, 23, 24).

### 2.4 Immunometabolic reprogramming and reduced treatment tolerance

Gastric cancer-related inflammation and malnutrition may also reduce tolerance to anticancer therapy through immunometabolic reprogramming (21, 25). Within the tumor microenvironment, tumor cells, immune cells, and stromal cells compete for glucose, amino acids, and lipid substrates, while hypoxia, lactate accumulation, and mitochondrial dysfunction may impair effector T-cell proliferation, cytotoxic activity, and cytokine secretion (21, 25). Under systemic inflammatory conditions, immunosuppressive components, including myeloid-derived suppressor cells, regulatory T cells, and M2-like macrophages, increase and further weaken antitumor immune responses (21, 25). In patients receiving chemotherapy, immunotherapy, or combination therapy, malnutrition and sarcopenia not only indicate insufficient energy reserves but also suggest reduced immune competence, impaired tissue repair capacity, and poorer drug tolerance. Clinically, these patients are more likely to experience treatment interruption, dose delay, infection, delayed postoperative recovery, and impaired quality of life (4, 24, 26). Therefore, nutritional intervention in gastric cancer should not be limited to caloric supplementation, but should also address inflammation control, muscle preservation, functional reserve, and treatment continuity (4).

**Figure 1:**
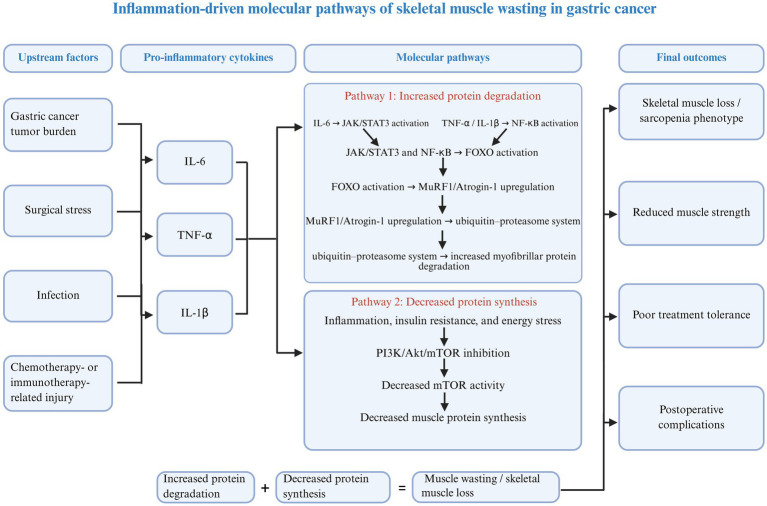
Inflammation-driven molecular pathways of skeletal muscle wasting in gastric cancer. A schematic overview of cytokine-mediated protein degradation and impaired protein synthesis in skeletal muscle. IL-6, interleukin-6; JAK/STAT3, Janus kinase/signal transducer and activator of transcription 3; TNF-*α*, tumor necrosis factor-alpha; IL-1β, interleukin-1 beta; NF-κB, nuclear factor kappa B; FOXO, forkhead box O; MuRF1, muscle RING finger protein 1; PI3K, phosphoinositide 3-kinase; Akt, protein kinase B; mTOR, mechanistic target of rapamycin. Created with BioRender.com.

Overall, gastric cancer-associated muscle wasting is not caused solely by inadequate intake, but is jointly driven by inflammatory signaling, enhanced protein degradation, and impaired protein synthesis (8, 21). Pro-inflammatory cytokines such as IL-6 and TNF-*α* may upregulate MuRF1 and Atrogin-1 through JAK/STAT3, NF-κB, and FOXO-related pathways, thereby enhancing ubiquitin–proteasome system-mediated muscle protein degradation. At the same time, suppression of PI3K/Akt/mTOR signaling may reduce the efficiency of muscle protein synthesis (8, 21). Together, these processes contribute to skeletal muscle loss, reduced muscle strength, and decreased treatment tolerance. The main molecular mechanisms are shown in Figure 1 (8, 21).

## 3 Nutritional assessment pathway for patients with gastric cancer

Gastric cancer-related malnutrition and sarcopenia are dynamic conditions that evolve with disease progression and treatment (4, 7). Inadequate intake often marks the onset of nutritional risk, while systemic inflammation and abnormal muscle protein metabolism may accelerate nutritional deterioration. Immunometabolic reprogramming may further affect treatment tolerance and clinical outcomes (4, 5, 7, 9). Therefore, nutritional assessment in gastric cancer should not rely solely on body weight, BMI, or albumin. Instead, it should integrate standardized nutritional risk screening with GLIM-based diagnosis, body composition analysis, assessment of muscle strength and physical performance, inflammatory markers, and treatment phase (4, 7, 10).

A feasible clinical pathway is to first identify nutritional risk using tools such as Nutritional Risk Screening 2002 (NRS 2002), Patient-Generated Subjective Global Assessment (PG-SGA), Subjective Global Assessment (SGA), or Mini Nutritional Assessment (MNA), and then apply the GLIM criteria to diagnose malnutrition and grade its severity (4, 10). CT-based body composition analysis and functional testing can subsequently be used to identify high-risk phenotypes, including low muscle mass, myosteatosis, sarcopenic obesity, and functional impairment (5, 7, 24, 27). In parallel, markers such as C-reactive protein (CRP), neutrophil-to-lymphocyte ratio (NLR), modified Glasgow Prognostic Score (mGPS), prognostic nutritional index (PNI), and controlling nutritional status (CONUT) may help characterize inflammatory burden and the risk of complications (9). This pathway translates the complex biological basis of nutritional deterioration into a clearer clinical assessment framework and provides a basis for defining nutritional requirements, selecting support strategies, and monitoring intervention response (4, 10).

### 3.1 Nutritional risk screening as the starting point of nutritional management

Nutritional management in patients with gastric cancer should begin with nutritional risk screening. During disease progression and treatment, reduced intake, early satiety, pyloric obstruction, treatment-related gastrointestinal toxicity, postoperative digestive dysfunction, and systemic inflammation may all place patients at high nutritional risk (3, 4). Therefore, before determining the intensity and route of nutritional support, it is necessary to clarify whether further assessment and intervention are warranted (4).

Tools such as NRS 2002, PG-SGA, SGA, and MNA or Mini Nutritional Assessment-Short Form (MNA-SF) can be used for nutritional screening or assessment in patients with gastric cancer. NRS 2002 is more suitable for rapid screening in hospitalized patients. PG-SGA is better aligned with the characteristics of patients with cancer, as it captures body weight changes, reduced intake, nutrition-related symptoms, and functional status. SGA can be used for global nutritional assessment, whereas MNA or MNA-SF is more appropriate for older patients (4, 28, 29). The shared value of these tools lies in identifying patients who require further assessment and intervention before marked weight loss or severe complications occur.

Importantly, a positive screening result does not equate to a diagnosis of malnutrition. Screening tools mainly indicate the presence of nutritional risk, but they do not fully capture reduced muscle mass, impaired muscle quality, inflammatory burden, or treatment-related metabolic changes (10, 30). Some patients with gastric cancer may already have skeletal muscle depletion or sarcopenic obesity despite a normal or elevated BMI. Others may present with inadequate intake, increased inflammation, and reduced treatment tolerance before a marked decline in serum albumin becomes evident (7, 30). Therefore, patients with positive screening results or a high clinical suspicion of nutritional risk should undergo further evaluation, including malnutrition diagnosis, body composition analysis, functional assessment, and assessment of inflammatory status (7, 10, 30).

### 3.2 GLIM criteria for the diagnosis of malnutrition

After nutritional risk screening, the GLIM criteria can be used to diagnose malnutrition (31). The GLIM framework combines phenotypic and etiologic criteria. Phenotypic criteria include unintentional weight loss, low BMI, and reduced muscle mass, whereas etiologic criteria include reduced intake or impaired absorption, as well as disease burden or inflammatory status (31). In general, the diagnosis of malnutrition requires at least one phenotypic criterion and one etiologic criterion (31).

The GLIM criteria are well aligned with the characteristics of malnutrition in patients with gastric cancer. Anorexia, early satiety, obstruction, nausea and vomiting, and treatment-related mucosal injury may lead to reduced intake. Tumor burden, *Helicobacter pylori*-related inflammation, surgical trauma, infection, and responses to systemic therapy may reflect disease burden or inflammatory status. CT-based body composition analysis and functional assessment can further help confirm reduced muscle mass and functional decline (10, 18, 32). Compared with approaches based solely on BMI, body weight, or serum albumin, the GLIM framework incorporates inadequate intake, inflammatory burden, and muscle phenotype within a single diagnostic structure, making it more consistent with the clinical features of malnutrition in gastric cancer (10, 18, 32, 33).

The use of the GLIM criteria may also improve comparability across studies. Previous studies have used heterogeneous definitions of malnutrition and sarcopenia, making it difficult to compare outcomes and synthesize evidence (31, 34, 35). A relatively standardized diagnostic framework may help clarify the prevalence and severity of malnutrition in patients with gastric cancer, as well as its associations with postoperative complications, treatment tolerance, and prognosis.

### 3.3 Body composition and functional assessment for detecting occult muscle depletion

After screening and diagnosis, body composition and functional assessment can further identify high-risk nutritional phenotypes (7, 36). In patients with gastric cancer, changes in body weight may not parallel muscle loss, particularly in those with sarcopenic obesity. In such cases, excess fat mass may mask skeletal muscle depletion, making BMI an unreliable indicator of muscle reserves (37). Abdominal CT is routinely used for diagnosis, staging, and treatment response assessment in gastric cancer. Therefore, assessment of skeletal muscle area, skeletal muscle index (SMI), skeletal muscle density (SMD), and myosteatosis at the third lumbar vertebra level is highly accessible and has potential clinical translational value (5, 36, 38). A low SMI usually indicates insufficient muscle reserves, whereas low SMD often reflects intramuscular fat infiltration. Unlike initial screening, CT-based body composition analysis is better positioned as an in-depth assessment after nutritional screening and GLIM-based diagnosis, supporting nutritional phenotype stratification and prediction of complication risk (5, 24, 36, 38).

However, muscle mass alone does not fully represent functional reserve. Functional indicators, including handgrip strength, gait speed, the timed up-and-go test, and performance status scores, can complement body composition measures by reflecting muscle strength, mobility, and overall functional capacity. In patients undergoing radical gastrectomy, neoadjuvant therapy, or systemic treatment, reduced muscle strength and slower gait speed may indicate an increased risk of delayed postoperative recovery, infection, falls, dose reduction, or treatment interruption. Therefore, body composition assessment should be combined with functional evaluation, rather than relying on a single imaging parameter to determine nutritional risk (39–41).

### 3.4 Inflammatory and laboratory markers as complementary dimensions of nutritional assessment

Inflammatory and laboratory markers can provide complementary information for nutritional risk stratification in patients with gastric cancer. Indicators such as CRP, interleukin-6 (IL-6), NLR, platelet-to-lymphocyte ratio (PLR), mGPS, PNI, and CONUT reflect inflammatory burden, immunonutritional status, and prognostic risk from different perspectives (9, 42–44). Integrating these markers with nutritional screening, GLIM-based diagnosis, and body composition analysis may help identify patients with inflammation-driven malnutrition and a higher risk of rapid muscle loss (4, 31).

However, these markers should not be interpreted in isolation from the clinical context. Albumin and prealbumin are readily influenced by inflammation, liver function, fluid status, infection, and surgical stress, and therefore should not be used alone to diagnose malnutrition. A decline in albumin during the perioperative period or systemic therapy may reflect the acute-phase response or inflammatory burden rather than insufficient protein intake. Although composite indices such as PNI and CONUT may support risk stratification, they cannot replace nutritional risk screening or GLIM-based diagnosis. A more appropriate approach is to use them as supportive indicators within a multidimensional assessment framework to reflect the degree of catabolic activity and the risk of complications (4, 31).

### 3.5 Dynamic assessment throughout the gastric cancer care continuum

The nutritional status of patients with gastric cancer changes across treatment phases, and assessment should be updated accordingly (3, 4). At initial diagnosis, nutritional risk screening, diagnosis of malnutrition, symptom assessment, and evaluation of baseline functional reserve should be completed. During neoadjuvant therapy or systemic treatment, monitoring should focus on dietary intake, gastrointestinal toxicity, body weight changes, the rate of muscle loss, inflammatory markers, and treatment adherence. In the perioperative period, attention should be paid to surgical stress, early postoperative recovery of oral intake, infectious complications, and restoration of gastrointestinal function (3, 12). Long-term follow-up after gastrectomy should also address weight recovery, changes in muscle mass, anemia, vitamin B12 deficiency, iron deficiency, calcium and vitamin D insufficiency, bone health, and quality of life (45–47).

**Figure 2:**
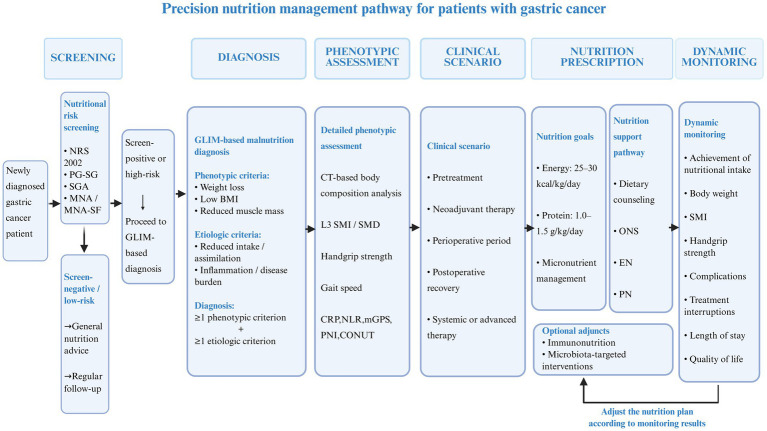
Precision nutrition management pathway for patients with gastric cancer. A stepwise framework integrating nutritional screening, GLIM-based diagnosis, phenotypic assessment, nutritional prescription, route selection, and dynamic monitoring. NRS 2002, Nutritional Risk Screening 2002; PG-SGA, Patient-Generated Subjective Global Assessment; SGA, Subjective Global Assessment; MNA, Mini Nutritional Assessment; MNA-SF, Mini Nutritional Assessment-Short Form; GLIM, Global Leadership Initiative on Malnutrition; BMI, body mass index; CT, computed tomography; L3, third lumbar vertebra; SMI, skeletal muscle index; SMD, skeletal muscle density; CRP, C-reactive protein; NLR, neutrophil-to-lymphocyte ratio; mGPS, modified Glasgow Prognostic Score; PNI, prognostic nutritional index; CONUT, controlling nutritional status; ONS, oral nutritional supplements; EN, enteral nutrition; PN, parenteral nutrition. Created with BioRender.com.

For patients with advanced or metastatic gastric cancer, assessment should further consider symptom burden, gastrointestinal patency, expected survival, the planned anticancer treatment strategy, and patient goals (3, 4). At this stage, nutritional intervention should not aim solely to increase intake, but should also focus on maintaining function, relieving symptoms, preserving treatment continuity, and improving quality of life (4). Overall, nutritional assessment in gastric cancer should form a continuous clinical decision-making pathway: screening identifies nutritional risk; the GLIM criteria confirm malnutrition; body composition and functional assessment evaluate muscle reserve and treatment vulnerability; inflammatory and laboratory markers indicate catabolic burden; and treatment phase determines the timing of intervention, the choice of support pathway, and the focus of monitoring. Based on this rationale, the key assessment, intervention, and monitoring nodes are integrated into a precision nutrition management flowchart for patients with gastric cancer (Figure 2) (3, 4, 12, 31).

## 4 Nutritional requirements and selection of support pathways in patients with gastric cancer

After nutritional assessment, the next step is to translate risk stratification into a specific nutritional prescription. For patients with gastric cancer, nutritional prescription should not be limited to determining whether nutritional risk is present; it should also define energy and protein targets, micronutrient management, and the route of nutritional delivery. The formulation of a nutritional plan should take into account treatment phase, oral intake capacity, gastrointestinal function, changes in body composition, anticancer treatment plans, and comorbidities. The goal is not only to correct intake deficits, but also to preserve skeletal muscle reserves, support treatment tolerance, promote postoperative recovery, and minimize nutrition-related complications whenever possible (4, 12).

In clinical practice, energy requirements are commonly estimated at 25–30 kcal/kg/day, and protein targets are generally set at 1.0–1.5 g/kg/day. For patients with sarcopenia, marked weight loss, postoperative recovery needs, hypercatabolism, or infection, a higher level of protein intake should be prioritized when renal function is adequate and gastrointestinal tolerance permits (4, 12). In patients with prolonged inadequate intake, severe wasting, electrolyte disturbances, or a risk of refeeding syndrome, high energy provision should not be initiated immediately. Instead, nutritional support should start at a low dose and be increased gradually, with concurrent monitoring of electrolytes, blood glucose, and fluid balance (48). Nutritional requirements, micronutrient management, and support pathway selection in patients with gastric cancer are summarized in Table 1.

**Table 1:** Nutritional requirements, micronutrient management, and route selection in patients with gastric cancer.

Item	Recommendation/dose	Applicable scenarios	Monitoring and precautions	References
Energy	25–30 kcal/kg/day; adjust according to clinical status	Perioperative period, systemic therapy, postoperative recovery	Monitor intake achievement, body weight, tolerance, and avoid overfeeding	(4, 12)
Protein	1.0–1.5 g/kg/day; 1.2–1.5 g/kg/day in sarcopenia or hypercatabolic states	Low SMI, sarcopenia, postoperative recovery, infection, ongoing weight loss	Adjust according to liver and renal function, SMI, handgrip strength, and gait speed	(4, 12)
Vitamin B12	Long-term or lifelong supplementation after total gastrectomy; 1,000 micrograms intramuscularly every 1–3 months, or oral ≥ 1,000 micrograms	Total gastrectomy, proximal gastrectomy, B12 deficiency, anemia, or neurological symptoms	Monitor complete blood count and serum B12; consider MMA or homocysteine when needed	(98)
Iron	Elemental iron 40–65 mg once daily or every other day; use intravenous iron if ineffective or poorly tolerated	Iron deficiency, iron-deficiency anemia, chronic blood loss, impaired absorption after gastrectomy	Monitor hemoglobin, ferritin, TSAT, and CRP; separate iron from calcium supplements	(99)
Folate	1–5 mg/day for confirmed deficiency, usually for 3–4 months	Inadequate intake, megaloblastic anemia, poor intake during chemotherapy	Exclude vitamin B12 deficiency before folate supplementation	(98)
Vitamin D	800–2,000 IU/day; adjust according to serum 25(OH)D	Vitamin D insufficiency, low bone mass, older age, total gastrectomy	Monitor 25(OH)D, calcium, phosphorus, and PTH	(46)
Calcium	Total intake 1,000–1,200 mg/day; preferably given in divided doses	Low bone mass, total or proximal gastrectomy, inadequate intake	Separate from iron supplementation; monitor serum calcium, renal function, and bone mineral density	(46, 100)
Zinc/selenium	Avoid routine high-dose supplementation; short-term targeted supplementation if deficiency is confirmed	Chronic diarrhea, long-term EN/PN, poor wound healing, taste disturbance	Avoid long-term excessive intake; reassess trace elements when indicated	(69)
Thiamine	100 mg before starting nutrition in patients at risk of refeeding syndrome; continue 100 mg/day for 5–7 days	Prolonged fasting, severe malnutrition, hypophosphatemia, hypokalemia, or hypomagnesemia	Monitor phosphorus, potassium, magnesium, glucose, and fluid balance	(48)
Dietary counseling	Oral intake first; small frequent meals, increased energy density, and high-quality protein	Patients able to eat orally but with inadequate intake	Assess early satiety, reflux, diarrhea, dumping syndrome, and treatment-related symptoms	(4, 12)
ONS	Add when ordinary diet is insufficient	Preoperative nutritional risk, postoperative weight loss, inadequate oral intake	Monitor adherence, tolerance, and body weight	(65)
EN	Preferred when oral intake is inadequate and the gastrointestinal tract is functional	Inadequate intake, gastric outlet obstruction with functional distal bowel, delayed postoperative recovery	Monitor abdominal distension, diarrhea, aspiration, tube-related complications, and target achievement	(49)
PN	Use when EN is contraindicated or fails to meet requirements	Bowel obstruction, severe malabsorption, uncontrolled vomiting, contraindication to EN	Monitor infection, glucose, electrolytes, liver function, and fluid overload	(69)

ONS, oral nutritional supplements; EN, enteral nutrition; PN, parenteral nutrition; SMI, skeletal muscle index; TSAT, transferrin saturation; CRP, C-reactive protein; PTH, parathyroid hormone; MMA, methylmalonic acid; 25(OH)D, 25-hydroxyvitamin D. This table is intended for use after initial nutritional risk screening and, when indicated, GLIM-based malnutrition diagnosis and body composition/function assessment. The doses listed are commonly used adult reference ranges rather than fixed prescriptions. Actual supplementation should be individualized according to the type of surgery, dietary intake, gastrointestinal absorption, laboratory results, liver and renal function, treatment stage, and local drug formulations.

The route of nutritional support should be selected stepwise, following the principles of oral intake first and enteral nutrition first. For patients who can eat orally, dietary counseling should be the initial approach. When a regular diet is insufficient to meet nutritional requirements, oral nutritional supplements should be added. If the gastrointestinal tract is functional but oral intake remains inadequate, enteral nutrition should be considered. Parenteral nutrition should be reserved for situations in which enteral nutrition is not feasible or cannot meet target requirements (4, 49). The specific pathway should not be determined by a single nutritional marker, but should be dynamically adjusted according to the proportion of intake achieved, gastrointestinal patency, absorptive capacity, treatment phase, risk of complications, expected benefit, and patient preference (4, 49, 50).

## 5 Nutritional intervention strategies across different clinical stages of gastric cancer

### 5.1 Need for stage-specific nutritional intervention

Nutritional problems in patients with gastric cancer are not confined to a single phase of treatment but evolve throughout diagnosis, treatment, and follow-up. Before treatment, early identification of nutritional risk and correction of inadequate intake are needed to provide a better foundation for surgery or systemic therapy. During neoadjuvant therapy, chemotherapy-related gastrointestinal toxicity, weight loss, and muscle depletion become more prominent. In the perioperative period, nutritional optimization before surgery, early postoperative recovery of oral intake, and control of infectious complications should be addressed together. After gastrectomy, long-term management should focus on weight recovery, anemia, vitamin and mineral deficiencies, and bone health. In advanced or metastatic disease, nutritional intervention is directed more toward symptom relief, functional maintenance, and quality of life. Therefore, nutritional support in gastric cancer should not follow a fixed protocol, but should be adjusted according to treatment phase, nutritional risk, gastrointestinal function, inflammatory status, body composition changes, and patient goals (1, 4).

### 5.2 Pretreatment phase

The pretreatment phase is a key window for shifting nutritional intervention earlier in the care pathway. At initial diagnosis or before surgery, chemotherapy, or immunotherapy, patients should undergo nutritional risk screening, GLIM-based diagnosis, body composition analysis, and assessment of muscle strength and physical performance. These evaluations should be combined with markers such as CRP, NLR, albumin, PNI, or CONUT to assess inflammatory burden and nutritional risk (4, 9, 10). For patients with unintentional weight loss, inadequate intake, low muscle mass, myosteatosis, sarcopenic obesity, high inflammatory burden, or advanced age with comorbidities, dietary counseling, oral nutritional supplements, or enteral nutrition should be initiated early, with the intensity of support determined according to gastrointestinal function and the planned treatment strategy. The goal at this stage is not simply to increase body weight, but to improve achievement of energy and protein targets, stabilize muscle reserves as much as possible, and prevent further nutritional depletion before surgery or systemic therapy begins (5).

### 5.3 Neoadjuvant therapy phase

During neoadjuvant therapy, nutritional management should focus on maintaining planned treatment delivery while minimizing muscle loss (3, 51). Patients with locally advanced gastric cancer undergoing neoadjuvant chemotherapy or perioperative chemotherapy may reduce food intake because of nausea, vomiting, diarrhea, oral or gastrointestinal mucositis, taste changes, anorexia, and fatigue, which may subsequently lead to weight loss, skeletal muscle depletion, and even treatment delay or interruption (51, 52). During this phase, dietary intake, body weight changes, gastrointestinal toxicity, inflammatory markers, muscle mass, and functional status should be monitored continuously (51, 53). When a regular diet is insufficient to meet nutritional requirements, oral nutritional supplements or enteral nutrition should be introduced promptly (4). For patients with sarcopenia, low SMI, or persistent weight loss, protein intake should be intensified on the basis of adequate energy provision, and moderate exercise or functional training may be incorporated according to individual tolerance (1, 4). It should be noted that nutritional intervention during neoadjuvant therapy primarily aims to support treatment tolerance and preserve functional capacity, whereas its effects on tumor regression or long-term survival require confirmation in higher-quality studies (51, 54).

### 5.4 Perioperative phase

The perioperative period is one of the most clinically actionable stages for nutritional intervention in gastric cancer (3, 12). Radical gastrectomy, particularly total gastrectomy combined with D2 lymphadenectomy, can induce substantial surgical stress, systemic inflammatory responses, and transient immunosuppression. In patients with pre-existing malnutrition or sarcopenia, the risks of postoperative infection, pulmonary complications, anastomosis-related complications, delayed recovery, and prolonged hospital stay may be further increased (55, 56). Therefore, perioperative nutritional management should be integrated into Enhanced Recovery After Surgery (ERAS) pathways. Patients with malnutrition or high nutritional risk should receive preoperative optimization, unnecessary fasting should be minimized, and oral or enteral nutrition should be resumed as early as possible after surgery according to the recovery of gastrointestinal function (12, 57). Immunonutrition formulas containing arginine, *ω*-3 PUFAs, and nucleotides have been investigated in high-risk patients undergoing gastrointestinal cancer surgery and may help reduce infectious complications and shorten hospital stay. However, their effects on non-infectious complications and long-term survival remain inconsistent, suggesting that immunonutrition is more appropriately used as an adjunctive strategy in selected high-risk patients (58–60).

### 5.5 Postoperative recovery phase

Postoperative recovery is not limited to short-term nutritional support before discharge, but also includes long-term follow-up after gastrectomy (61). After gastrectomy, patients often experience prolonged inadequate intake due to reduced gastric capacity, early satiety, reflux, dumping syndrome, diarrhea, anorexia, and changes in eating patterns, and weight loss may persist for several months or even longer than 1 year (61, 62). After total or proximal gastrectomy, particular attention should also be paid to vitamin B12 deficiency, iron-deficiency anemia, folate insufficiency, calcium and vitamin D deficiency, and bone loss (45, 63, 64). During follow-up, body weight, dietary intake, complete blood count, ferritin, transferrin saturation, vitamin B12, folate, 25-hydroxyvitamin D, calcium and phosphorus metabolism, and bone health should be assessed regularly. For patients with inadequate intake or persistent weight loss after discharge, oral nutritional supplements may be added to dietary counseling (4, 65). If long-term oral intake remains insufficient, enteral nutrition may be considered according to gastrointestinal function, with supplemental parenteral nutrition used when necessary (4, 49). At this stage, the goal should extend beyond short-term caloric supplementation to include body weight and muscle maintenance, correction of micronutrient deficiencies, functional recovery, and improvement in quality of life. Common micronutrient deficiencies after gastrectomy and the corresponding principles of supplementation are summarized in Table 1.

### 5.6 Advanced or metastatic disease

Nutritional intervention in patients with advanced or metastatic gastric cancer should be closely aligned with symptom burden, treatment goals, and patient preferences (1, 4). At this stage, tumor-related anorexia, cachexia, gastric outlet obstruction, peritoneal metastasis, malignant ascites, bowel obstruction, recurrent vomiting, diarrhea, and systemic treatment-related toxicities are common (66, 67). For patients who are still receiving anticancer therapy and are expected to benefit from nutritional support, nutritional intervention may help maintain physical strength, reduce treatment interruptions, and improve symptoms and quality of life (4, 68). When the gastrointestinal tract is patent and tolerated, dietary modification, oral nutritional supplements, or enteral nutrition should be prioritized. In patients with gastric outlet obstruction but preserved distal intestinal function, management may include endoscopic stenting, gastrointestinal decompression, or jejunal feeding access (66). If irreversible bowel obstruction, severe malabsorption, or inability to implement enteral nutrition is present, parenteral nutrition may be considered after careful assessment of prognosis, complication risk, and patient goals (67, 69). For patients at the terminal stage or with very limited expected survival, nutritional support should avoid overmedicalization and shift its focus toward comfort care, symptom relief, and respect for patient preferences (70).

## 6 Immunonutrition and microbiota-targeted interventions

### 6.1 Immunonutrition

**Figure 3:**
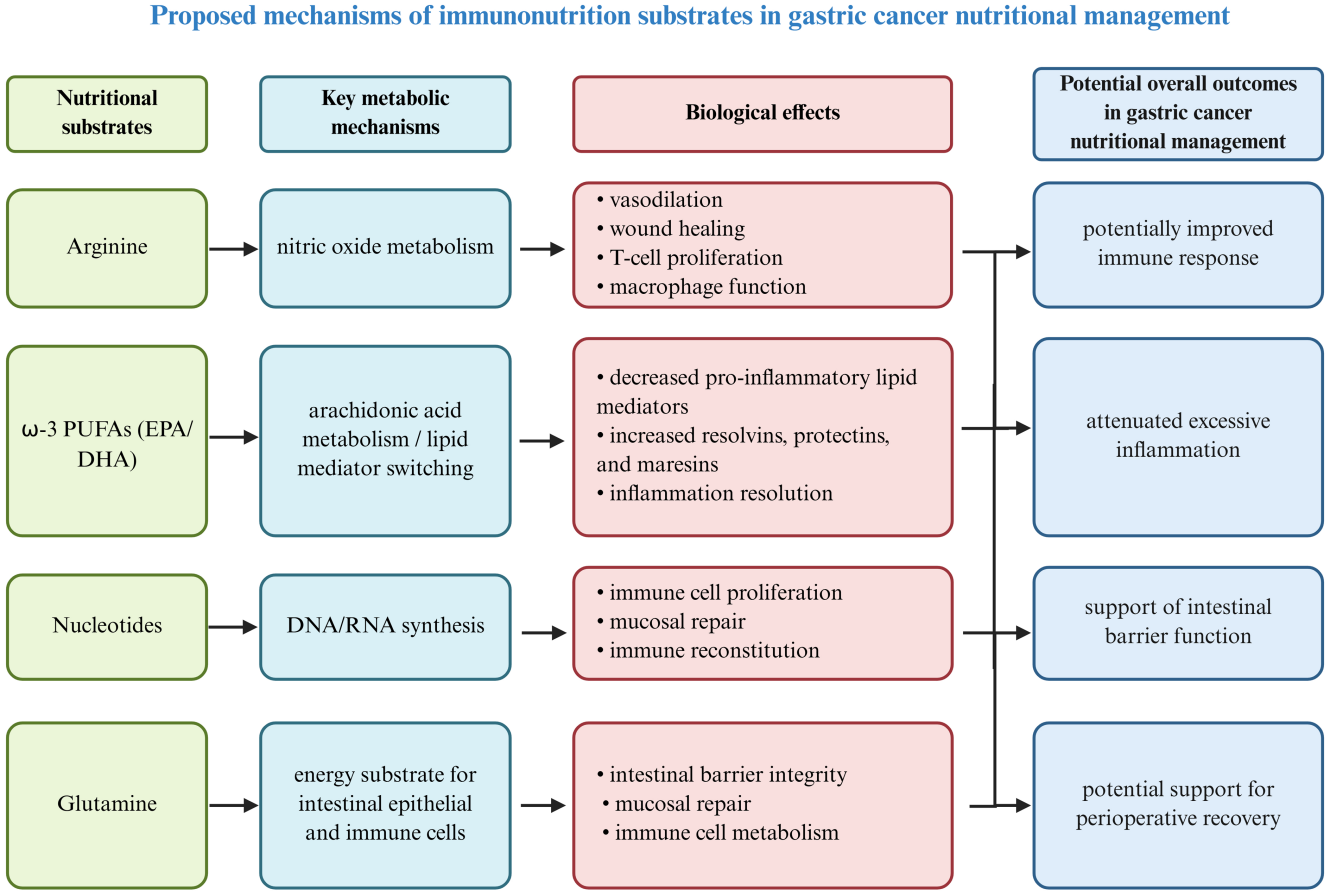
Proposed mechanisms of immunonutrition substrates in gastric cancer nutritional management. A schematic summary of the immunometabolic effects of arginine, omega-3 PUFAs, nucleotides, and glutamine. PUFAs, polyunsaturated fatty acids; EPA, eicosapentaenoic acid; DHA, docosahexaenoic acid; DNA, deoxyribonucleic acid; RNA, ribonucleic acid; NO, nitric oxide. Created with BioRender.com.

Immunonutrition does not simply involve increasing energy or protein provision. Rather, it refers to the supplementation of nutritional substrates with immunomodulatory or anti-inflammatory properties, including arginine, *ω*-3 polyunsaturated fatty acids, nucleotides, and glutamine, on the basis of standard nutritional support (4, 12). Under conditions such as surgical trauma, infection, anticancer therapy, or hypercatabolism, these substrates may participate in the regulation of host immune responses, intestinal barrier function, and tissue repair (12, 71). As a conditionally essential amino acid, arginine may influence vasodilation, wound healing, T-cell proliferation, and macrophage activation through nitric oxide synthesis pathways (71, 72). *ω*-3 polyunsaturated fatty acids, including EPA and DHA, can modulate arachidonic acid metabolism, reduce the production of pro-inflammatory lipid mediators, and promote the formation of lipid mediators involved in inflammation resolution (73). Nucleotides may support lymphocyte proliferation, intestinal mucosal repair, and immune responses (4, 12). Glutamine is an important energy substrate for intestinal epithelial cells and immune cells and may help maintain intestinal mucosal barrier integrity (71, 72). Overall, the effects of immunonutrients extend beyond the provision of energy or nitrogen sources and involve processes such as nitric oxide metabolism, lipid mediator switching, immune cell proliferation, and energy metabolism within the intestinal barrier. The main mechanisms are shown in Figure 3 (4, 12, 71, 72).

### 6.2 Microbiota-targeted interventions

Unlike immunonutrition, which mainly focuses on specific nutritional substrates, microbiota-targeted interventions emphasize host–microbe interactions (14). Approaches such as probiotics, prebiotics, synbiotics, dietary modulation, and fecal microbiota transplantation may influence the mucosal barrier, inflammatory responses, immune status, and treatment-related adverse events by modifying the microbiota and its metabolites (14, 74). Gastric cancer development and progression are closely associated with alterations in the gastric microbiota. *Helicobacter pylori* is a well-established initiating factor, while the role of non-*H. pylori* microbiota in gastric cancer progression has received increasing attention (14, 75). Changes in microbial composition have been observed in gastric cancer tissues, gastric juice, oral samples, and fecal samples, and some studies have reported enrichment of taxa such as Streptococcus and Fusobacterium in gastric cancer tissues (14, 75–77). However, substantial differences in sample types, population characteristics, dietary factors, sequencing methods, and analytical pipelines across studies mean that current findings remain inconsistent (14).

At the clinical level, probiotics, prebiotics, and synbiotics may help improve treatment-related diarrhea, intestinal barrier injury, and inflammatory status. They may also participate in the regulation of host nutritional metabolism through short-chain fatty acids, mucosal barrier function, and immune cell activity (14, 74, 78). This approach has theoretical relevance for patients receiving chemotherapy, immunotherapy, or post-gastrectomy care, particularly in settings involving diarrhea, mucosal injury, and impaired intestinal barrier function (74, 78, 79). Nevertheless, existing studies vary considerably in bacterial strains, doses, intervention duration, and outcomes, and gastric cancer-specific evidence remains limited. Therefore, probiotics, prebiotics, and synbiotics are currently more appropriately regarded as adjunctive measures for nutritional support and symptom management, rather than as standard components of precision nutrition in gastric cancer (14, 79).

### 6.3 FMT and evidence limitations

The association between the gut microbiota and the efficacy of immune checkpoint inhibitors has made fecal microbiota transplantation (FMT) an important exploratory direction among microbiota-targeted interventions (74, 80). Existing evidence suggests that the gut microbiota may help regulate responses to PD-1/PD-L1 inhibitors by influencing immune processes such as antigen presentation, T-cell activation, myeloid-derived suppressor cells, and regulatory T cells (74, 80). In recent years, FMT has been preliminarily investigated in solid tumors resistant to immunotherapy. In some patients with refractory solid tumors, FMT combined with anti-PD-1 therapy has been associated with microbiota remodeling and signals of disease control (81–83). FMT has also been explored for refractory immune checkpoint inhibitor-related colitis, suggesting that it may help improve certain immune-related adverse events (84).

However, these findings should be interpreted with caution. Current evidence is derived mainly from patients with melanoma or mixed solid tumors, with limited sample sizes. Donor selection, routes of administration, safety, predictive biomarkers of efficacy, and long-term outcomes remain unclear (81–84). In patients with gastric cancer, the use of FMT to overcome immunotherapy resistance, or as a nutrition–immune modulation strategy, remains exploratory. Similarly, there is insufficient evidence to support the selection of probiotics, synbiotics, or FMT based on a single microbiota test. Future studies should be conducted in gastric cancer-specific cohorts and should integrate tumor molecular features, microbiota composition, microbial metabolites, body composition, and immunotherapy outcomes to define the populations most likely to benefit, the underlying mechanisms, and the safety boundaries of microbiota-targeted interventions (14, 74).

## 7 Molecular features and evidence limitations of precision nutrition

### 7.1 Molecular classification and treatment-related biomarkers in gastric cancer

Gastric cancer is not a biologically homogeneous disease. Different histological types and molecular subtypes vary in patterns of invasion, treatment response, and prognosis (1, 3). The Cancer Genome Atlas classified gastric cancer into Epstein–Barr virus-positive, microsatellite instability, chromosomal instability, and genomically stable subtypes. Epstein–Barr virus-positive and MSI-H/dMMR tumors are generally characterized by prominent immune infiltration; the chromosomal instability subtype is frequently associated with amplification of receptor tyrosine kinase-related genes; and the genomically stable subtype is more commonly observed in diffuse-type gastric cancer and is linked to abnormal cell adhesion and aggressive biological behavior (1, 3). In recent years, biomarkers such as HER2, PD-L1 CPS, MSI/dMMR, EBV, CLDN18.2, and FGFR2b have increasingly been used to guide targeted therapy, immunotherapy selection, and clinical trial stratification in advanced gastric cancer (3, 85–88). These molecular features help explain heterogeneity in treatment response, but at present they are mainly used for anticancer treatment decision-making and cannot be directly translated into nutritional prescriptions (4, 11).

### 7.2 Potential links between molecular features and nutritional phenotypes

From a mechanistic perspective, molecular features may indirectly influence nutritional risk through the immune microenvironment, systemic inflammation, patterns of disease progression, and treatment-related toxicity profiles (1, 4, 11). MSI-H/dMMR and Epstein–Barr virus-positive gastric cancers are often characterized by higher levels of immune cell infiltration and immune checkpoint molecule expression, which may be associated with more active local immune responses and inflammatory status. Tumors of the chromosomal instability subtype frequently involve alterations in pathways such as HER2, EGFR, FGFR2, and MET, and the corresponding treatment strategies and drug-related adverse events may affect appetite, gastrointestinal tolerance, and the need for nutritional support. Genomically stable tumors, as well as diffuse-type, poorly differentiated, or signet ring cell-associated gastric cancers, are more prone to gastric wall infiltration, peritoneal dissemination, gastrointestinal obstruction, and reduced intake, thereby increasing the risk of weight loss, protein depletion, and sarcopenia (1, 3, 11, 53). Although these associations are biologically plausible, the current evidence remains largely indirect and does not yet demonstrate that a specific molecular subtype necessarily corresponds to a stable nutritional phenotype or a predictable response to nutritional intervention (4, 11).

### 7.3 Current boundaries of molecularly guided nutritional prescription

Based on current evidence, nutritional intervention in gastric cancer cannot be determined directly by a single molecular marker (4, 11). Results for HER2, PD-L1, MSI/dMMR, EBV, CLDN18.2, or FGFR2b may help characterize tumor biology, guide anticancer treatment selection, and identify potential risks of treatment-related adverse events. However, they do not indicate that a given subgroup of patients must receive a specific nutritional substrate, immunonutrition formula, microbiota-targeted interventions, or fixed nutritional support pathway (3, 4, 11, 85–88). Nutritional prescriptions should still be based on clinical nutritional assessment, including nutritional risk screening, GLIM-based diagnosis, body composition and functional assessment, inflammatory status, intake capacity, gastrointestinal function, and treatment phase (4, 89). Prematurely equating molecular features with the basis for nutritional prescription may overstate the current evidence and weaken the foundational role of standardized nutritional assessment (4, 11).

### 7.4 Future research directions

Future research should address not only whether molecular features can explain differences in nutritional risk among patients with gastric cancer, but also whether they can predict which patients are most likely to benefit from nutritional intervention (4, 11). Ideally, such studies should be based on prospective gastric cancer-specific cohorts and should collect, in parallel, tumor molecular subtypes, treatment regimens, nutritional risk screening results, GLIM-based diagnosis, CT-derived body composition measures, muscle strength and physical performance, inflammatory markers, dietary intake, treatment-related toxicities, and clinical outcomes. Stratified analytical models should be predefined (4, 11, 53, 89). Particular attention should be paid to whether distinct molecular subtypes are associated with different inflammatory phenotypes and rates of muscle loss, whether changes in body composition affect tolerance to chemotherapy, targeted therapy, or immunotherapy, and whether patients with specific inflammatory or metabolic backgrounds are more likely to benefit from nutritional intervention (11, 53, 89). Only through prospective, multicenter studies that integrate mechanistic indicators with clinical endpoints can the true value of molecular information in precision nutrition for gastric cancer be determined, while avoiding the premature translation of early exploratory associations into clinical prescriptions.

## 8 Future technologies in precision nutrition for gastric cancer

The implementation of stratified precision nutrition in gastric cancer requires more stable and efficient assessment tools (53, 90). CT-based body composition analysis can objectively quantify skeletal muscle mass, muscle density, fat distribution, and myosteatosis, and is valuable for identifying sarcopenia, myosteatosis, and sarcopenic obesity (53, 91). However, conventional manual or semi-automated measurements are time-consuming and may be influenced by operator experience and inter-center variability (16, 90). Artificial intelligence and deep learning can automatically identify the third lumbar vertebra level, segment skeletal muscle and adipose tissue, and calculate skeletal muscle index, muscle density, and fat area (16, 90). If further standardized, these technologies may be used to screen high-risk nutritional phenotypes before surgery and to monitor muscle loss during neoadjuvant therapy, the perioperative period, and postoperative follow-up, making them particularly suitable for real-world studies and longitudinal surveillance (16, 90–92).

Beyond body composition analysis, radiomics and multi-omics integration may also help explain heterogeneity in nutritional risk among patients with gastric cancer (14, 93, 94). Radiomics can extract tumor morphology, texture, density, and spatial heterogeneity from routine imaging modalities such as CT, MRI, or PET-CT. When combined with muscle and fat parameters, these features may provide a more comprehensive picture of tumor burden, muscle reserve, and fat distribution (93, 95). Genomics, transcriptomics, proteomics, metabolomics, microbiomics, and peripheral inflammatory markers can further characterize tumor–host interactions at different biological levels (14, 94). Integrating these data with nutritional risk screening, GLIM-based diagnosis, achievement of intake targets, treatment-related toxicity, inflammatory markers, and treatment-relevant molecular biomarkers may improve the identification of nutrition-related risk phenotypes and support the development of predictive models (4, 91, 94).

A key future challenge is how to translate these data into dynamic risk models that can support clinical decision-making (93, 95, 96). Ideally, such models should be continuously updated during treatment and should integrate body weight changes, dietary intake, gastrointestinal symptoms, treatment toxicities, CT-derived body composition parameters, muscle strength, physical performance, inflammatory markers, nutrition-related laboratory indicators, molecular biomarkers, and microbiota data. These models could be used to predict the onset of malnutrition, progression of sarcopenia, postoperative complications, treatment dose delays, reduced treatment tolerance, and potential benefit from nutritional intervention (4, 91, 94, 96). In this sense, nutritional management should not wait until substantial weight and muscle loss have occurred; instead, patients should be identified earlier when their risk begins to increase. Nevertheless, these technologies are still mainly at the stage of risk stratification and model development. They cannot yet replace standardized nutritional screening, GLIM-based diagnosis, or clinical assessment, nor can they directly generate individualized nutritional prescriptions (4, 89). Future studies should rely on prospective, multicenter gastric cancer-specific cohorts, with harmonized definitions for body composition measurement, nutritional diagnosis, omics data collection, and clinical outcomes, followed by external validation and interventional studies to confirm their clinical utility (89, 95).

## 9 Evidence limitations and future directions

Current evidence suggests that standardized assessment and basic nutritional support remain the most reliable foundation of nutritional management in gastric cancer (4). The clinical pathway should begin with nutritional risk screening using tools such as NRS 2002, PG-SGA, SGA, and MNA. Patients with positive screening results or a high clinical suspicion of nutritional risk should then undergo GLIM-based diagnosis of malnutrition (4, 89). CT-based body composition analysis, together with assessment of muscle strength and physical performance, can further identify high-risk phenotypes such as low muscle mass, myosteatosis, sarcopenic obesity, and functional impairment (53). Markers including CRP, NLR, mGPS, PNI, and CONUT may provide additional information on inflammatory burden and prognostic risk (97). Nutritional intervention should remain centered on adequate energy and protein provision and should be adjusted according to intake, gastrointestinal function, inflammatory status, treatment phase, and changes in body composition. In terms of route selection, oral intake and enteral nutrition should be prioritized. Oral nutritional supplements should be added when a regular diet is insufficient; enteral nutrition should be used when oral intake remains inadequate but gastrointestinal function is preserved; and parenteral nutrition should be considered only when enteral nutrition is not feasible or cannot meet target requirements. Perioperative nutritional optimization, early postoperative recovery of oral or enteral nutrition, and adjunctive immunonutrition in patients at high nutritional risk are supported by evidence suggesting benefits for short-term recovery and reduction of infectious complications (12).

Compared with these relatively established measures, several precision-oriented strategies require more cautious interpretation. Biomarkers such as HER2, PD-L1, MSI/dMMR, EBV, CLDN18.2, and FGFR2b are useful for characterizing tumor biology and guiding anticancer treatment, but they cannot currently be used to directly determine nutritional prescriptions (3, 4). Whether stable associations exist between molecular subtypes and nutritional risk phenotypes, the rate of muscle loss, or response to nutritional intervention remains insufficiently supported by prospective evidence (11). Microbiota-targeted interventions face similar limitations. A single microbiota test is not sufficient to guide the selection of probiotics, prebiotics, or synbiotics, and the use of FMT to overcome immunotherapy resistance in gastric cancer or as a nutrition–immune modulation strategy is still largely extrapolated from studies in other tumors or mixed solid tumor populations (14). AI-assisted body composition analysis, multi-omics integration, and dynamic prediction models provide new tools for risk stratification, but they are still mainly used for research and model development. They cannot yet replace standard nutritional screening, GLIM-based diagnosis, or clinical nutritional assessment (89, 91).

Future research should address not only how to identify nutritional risk, but also which patients are most likely to benefit from specific nutritional strategies. Existing studies have important methodological limitations, including the predominance of single-center retrospective designs, inclusion of mixed tumor populations, heterogeneous definitions of malnutrition and sarcopenia, insufficient use of GLIM criteria, inconsistent CT measurement levels and SMI cut-offs, variability in intervention protocols and follow-up duration, and inadequate control of outcomes and confounding factors (11, 53, 89, 91). Prospective, multicenter gastric cancer-specific cohorts are needed, with harmonized definitions for nutritional screening, GLIM-based diagnosis, body composition measurement, functional assessment, and clinical outcomes. These studies should also collect dietary intake, treatment-related toxicities, inflammatory markers, molecular features, microbiota profiles, and metabolomic data in parallel (11, 14, 91). Only on this basis can the true value of body composition, inflammation, microbiota, and multi-omics information be more reliably determined for predicting nutritional risk, treatment tolerance, and benefit from nutritional intervention.

## 10 Conclusion

Malnutrition and sarcopenia in patients with gastric cancer occur throughout diagnosis, treatment, and follow-up. Their development is not solely attributable to inadequate intake, but is closely related to tumor burden, systemic inflammation, metabolic reprogramming, treatment-related toxicities, and altered digestion and absorption after gastrectomy. Based on current evidence, the most reliable foundation for nutritional management in gastric cancer remains standardized screening and clinical phenotype assessment. Nutritional status should first be defined through nutritional risk screening and the GLIM criteria, followed by the integration of body composition, functional status, inflammatory burden, and treatment phase to guide intervention strategies (4, 89). On this basis, setting appropriate energy and protein targets, correcting micronutrient deficiencies, and selecting dietary counseling, ONS, EN, or PN according to gastrointestinal function remain the most practical clinical pathway (4). Perioperative immunonutrition, long-term postoperative nutritional follow-up, and symptom-oriented support for patients with advanced disease should also be considered within the context of treatment phase and patient goals.

Precision nutrition provides a new direction for nutritional management in gastric cancer, but at the current stage, it should not be interpreted as a direct translation of molecular biomarkers into nutritional prescriptions (11). AI-assisted body composition analysis, multi-omics integration, microbiota-targeted interventions, molecular classification, and dynamic risk prediction models may help identify high-risk populations, explain interindividual differences, and optimize the timing of intervention. However, single markers such as HER2, PD-L1, MSI/dMMR, EBV, CLDN18.2, or FGFR2b are currently insufficient to determine specific nutritional substrates, immunonutrition formulas, or microbiota-targeted strategies (11, 14). Similarly, nutritional decision-making based on microbiota testing, FMT, or multi-omics models still requires validation in prospective studies (14). Therefore, precision nutrition in gastric cancer should currently be positioned as a stratified management model built on standardized nutritional assessment and guideline-recommended supportive strategies. Future research should use prospective, multicenter gastric cancer-specific cohorts with harmonized diagnostic criteria, measurement methods, and outcome definitions to further clarify the value of body composition, inflammation, microbiota, and multi-omics information in predicting nutritional risk, assessing treatment tolerance, and identifying patients most likely to benefit from nutritional intervention.
